# The impact of family alexithymia on the severity of restrictive eating disorders in adolescent patients

**DOI:** 10.1186/s13034-023-00692-x

**Published:** 2023-12-19

**Authors:** Francesca Marazzi, Marika Orlandi, Valentina De Giorgis, Renato Borgatti, Martina Maria Mensi

**Affiliations:** 1 Department of Brain and Behavioural Sciences, University of Pavia, Pavia, Italy; 2 Child Neurology and Psychiatry Unit, IRCCS Mondino Foundation, Pavia, Italy

**Keywords:** Alexithymia, Family, Restrictive eating disorders, Toronto Alexithymia Scale

## Abstract

**Background:**

Alexithymia is the inability to identify and describe one’s own emotions. Adolescents who suffer from Restrictive Eating Disorders (REDs) show a higher prevalence of alexithymia than the general population.

**Methods:**

The study explored the correlation between levels of alexithymia in mothers, fathers, and adolescents affected by REDs and patients’ ability to recognize their emotions. The study also aimed to evaluate if patients’ emotional distress can significantly impact the severity of their disorder and functioning measured by the Clinical Global Impression Scale - Severity (CGI-S) and the Children’s Global Assessment Scale (CGAS). We enrolled 67 families of adolescents affected by REDs. Parents and patients’ levels of alexithymia were assessed through the Toronto Alexithymia Scale (TAS-20). Spearman’s correlation shows a statistically significant correlation between mothers and patients’ levels of alexithymia.

**Results:**

Our findings also suggest that fathers and mothers’ TAS scores correlate with each other. However, there is no statistically significant relationship between the influence of the TAS scores of fathers and sons/daughters.

**Conclusions:**

In conclusion, mothers’ level of alexithymia could influence both fathers and patients’ difficulty in identifying and describing their own emotions. This relationship can be investigated further when considering externally oriented thinking. However, the severity of the disease and overall functioning do not appear to be affected by patients’ levels of alexithymia.

**Supplementary Information:**

The online version contains supplementary material available at 10.1186/s13034-023-00692-x.

## Introduction

Alexithymia has been defined as the difficulty and inability to recognize and describe one’s own emotions and feelings, along with concrete and externally oriented thinking [1]. It has been reported that people who presented alexithymia have impairments in emotion-processing and regulating abilities, as well as difficulties in recognizing their own physical and emotional symptoms [2]. Psychotherapists noted that alexithymia was a common characteristic observed among classic psychosomatic patients in whom therapy was unsuccessful [3]. Many studies reported how alexithymia is related to different mental disorders, for example, anxiety disorders [4], depression [5], substance abuse [6], and eating disorders (EDs) [7]. Research and reviews pointed out that alexithymia may have a role as a predictor of treatment outcomes in the population affected by anorexia nervosa (AN), in fact, the higher the alexithymia levels, the worse the outcomes [8, 9]. However, alexithymic features are not only limited to patients but they have been recorded in the general population: studies conducted in adults showed a prevalence of clinically significant alexithymia of about 10% [10, 11]. Currently, the Toronto Alexithymia Scale (TAS-20) is the most widely used instrument for assessing alexithymia, since multiple studies have shown it to have internal consistency, high test-retest reliability, construct validity, and criterion validity [12–14], despite being a self-administered questionnaire. The TAS-20 focuses on three main features: difficulties in recognizing and verbalizing emotions and externally oriented thinking [15].

We identified a heterogeneous clinical research group of patients affected by restrictive EDs that we call Restrictive Eating Disorders (REDs) group [16]. The REDs group includes AN, atypical anorexia (A-AN), avoidant/restrictive food intake disorder (ARFID), and other non-specified EDs with restrictive characteristics [17]. This group of EDs affects a predominantly young female population, with an increased prevalence during the last years, in adolescents from 15 to 19 years old [18].

Adolescents affected by REDs have a major risk for comorbid behavioural symptoms [19] and a higher prevalence of alexithymia than the general population [20]. Literature also stated that impairment in identifying and describing one’s emotions can be considered a fully-fledged transdiagnostic factor across the ED spectrum [21], with greater emotion regulation difficulties and reduced emotional awareness [22]. In addition, alexithymia has recently been proposed as a potential transdiagnostic therapeutic target for ED patients [23].

Despite the strong association between alexithymia and several mental health problems, the literature still struggles to explain the reasons why alexithymia features are more linked to some disorders than others. Numerous authors investigated family influence and childhood family factors [24–26]. Nonetheless, other researchers reported the presence of genetic influence on the alexithymia traits [27–29]. Considering this aspect, it is possible to notice how the three main features of alexithymia are related to families’ emotional functioning. In particular, impairment in identifying feelings has been related to dysfunctional family affective involvement, externally oriented thinking has been associated with deficient family behaviour control and impaired imagination has been linked to inadequate family problem-solving. This finding suggested that dysfunctional family functioning is implicated in the development of alexithymic characteristics in children [29].

Specifically, many studies investigated the emotional functioning of families where an adolescent suffers from RED, finding out how it can play a fundamental role in the evolution of the disorder and in the therapies that involve not only the young patient but his/her family as well [30]. For a very long time, the onset of ED in adolescents has been reported to be influenced by mothers [31]. This finding has led literature to investigate the relationship between mothers and daughters affected by REDs [32, 33]. Nonetheless, a correlation between dysfunctional family traits and sons/daughters with a diagnosis of REDs has been found [34]. This result underlined the importance of further study to explore the relationship between REDs and family functioning, assessing each family member.

Many authors demonstrated that family functioning significantly impacts treatment outcomes [35–37]. In particular, one study suggested how positive family relationships and patients’ interactive behaviour might correlate with lower RED severity [38]. Despite the significant link, literature focused more on individual alexithymia, rather than deeply exploring the relationship between families and patients, presenting fewer studies on alexithymia in parents of adolescent patients affected by REDs [39–41]. One previous study added that there may be a significant relationship between the young RED patients’ risk for alexithymia and the presence of a specific pattern of family interactive dysfunction [42]. Despite this evidence, the influence of parents’ alexithymia levels on young RED patients is still unclear.

Based on the above, the main goal of the study is to assess the alexithymic characteristics of adolescents affected by REDs and their mothers and fathers. We want to evaluate if the emotional distress of RED patients might be related to their parents’ emotional functioning. Specifically, we hypothesize to observe a statistically significant correlation between alexithymia levels of different family members. Finally, the other aim of the present study is to better explore the impact of adolescents’ impaired emotions-processing on their eating disorder severity. In particular, we want to investigate whether alexithymia levels could be related to the disease severity and patients’ global functioning.

## Methods

### Participants

Sixty-seven families of adolescent patients diagnosed with REDs were enrolled between March 2016 and March 2022 at the Child Neurology and Psychiatry Unit of the tertiary care IRCCS Mondino Foundation (Pavia, Italy) in a cross-sectional study. Patients were considered eligible for the study if they were 12–18 years old and if they had a diagnosis of REDs (including restrictive and binge-eating/purging subtypes of anorexia nervosa, atypical anorexia nervosa, other non-specified feeding or eating disorders with restrictive characteristics). Diagnoses were made following the Diagnostic and Statistical Manual of Mental Disorders (DSM-5) criteria [17] and they were confirmed using the DSM-based Kiddie Schedule for Affective Disorders and Schizophrenia (K-SADS-PL DSM-5) [43].

**Fig. 1:**
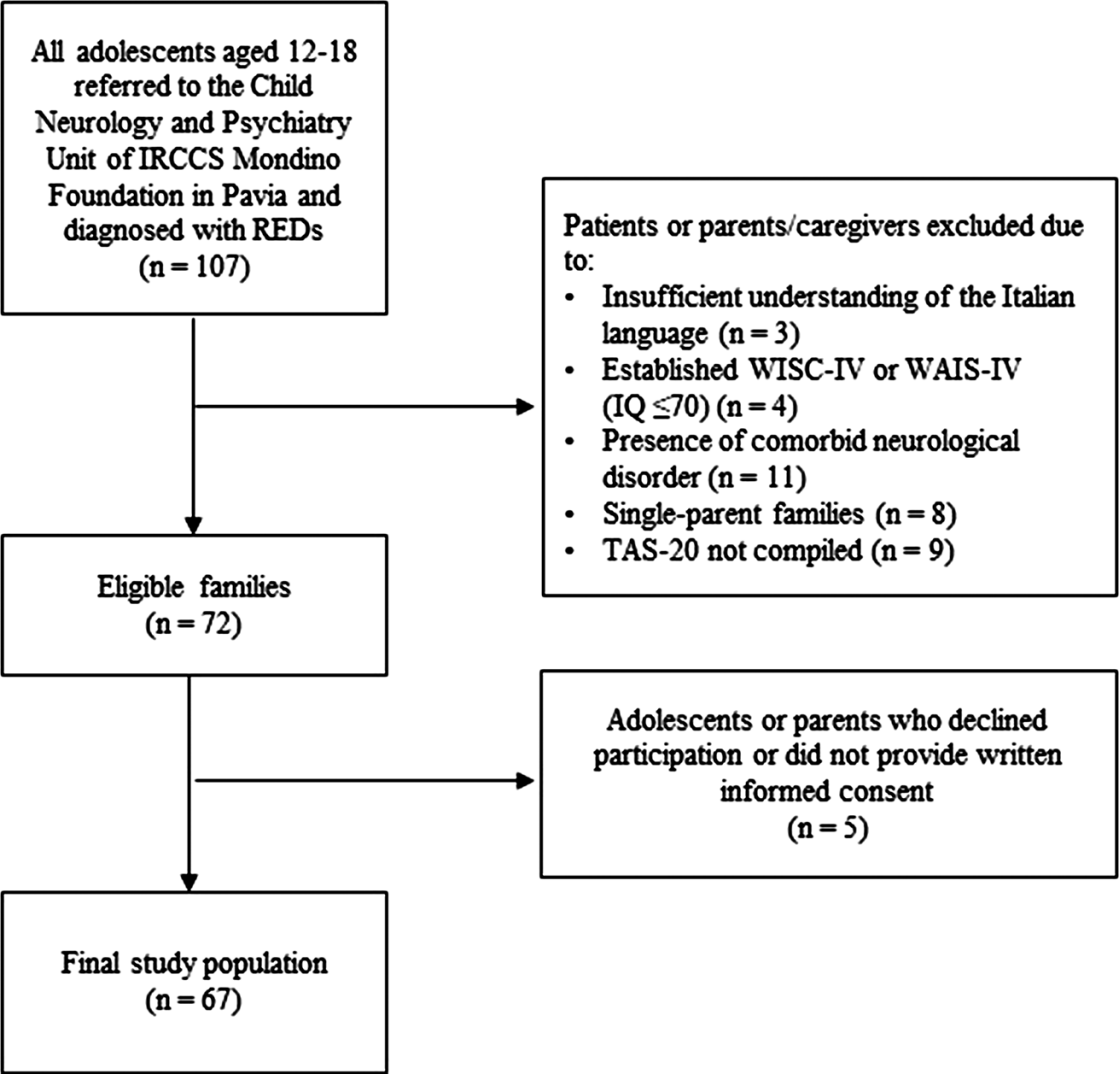
Study population flowchart

Patients were excluded from the study if they presented with comorbid neurological disorders or intellectual disability, which was confirmed by age-appropriate Wechsler Intelligence Scale (WISC-IV or WAIS-IV) [44, 45]. Single-parent families and individuals unable to well understand Italian were also considered ineligible for the present study. All the enrolled patients and their parents provided written informed consent to participate in the study (Fig. 1).

The study received the approval of the Ethics Committee of Policlinico San Matteo in Pavia (Protocol ID: P-20,170,016,006); it was performed according to the Reporting of studies Conducted using Observational Routinely collected health Data (RECORD) statement (Supplementary material). The authors assert that all procedures contributing to this work comply with the ethical standards of the relevant national and institutional committees on human experimentation and with the Helsinki Declaration of 1964 and its later amendments.

### Procedures

Patients and parents were interviewed by a trained child neuropsychiatrist about medical and family history, followed by psychiatric evaluation and a complete clinical neurologic examination. To evaluate the level of alexithymia, adolescent patients and their parents filled in the Toronto Alexithymia Scale (TAS-20) [12, 14] referring to themselves. The TAS is a 20-item self-administered questionnaire in which each item scored 1 (strongly disagree), 2 (disagree), 3 (neither agree nor disagree), 4 (agree), or 5 (strongly agree). It is possible to identify 3 subscales: difficulty identifying feelings (DIF, which includes items 1, 3, 6, 7, 9, 13, and 14); difficulty describing feelings (DDF, which include items 2, 4, 11, 12, and 17); externally oriented thinking (EOT, which include items 5, 8, 10, 15, 16, 18, 18, 19 and 20). The total alexithymia score is the sum of responses to all 20 items, while the score for each subscale factor is the sum of the responses to that subscale. The total score, variable from 20 to 100, is classified as follows:

- 20–51 = not alexithymic;
- 52–60 = borderline;
- 61–100 = alexithymic.

In addition, the same clinician compiled the Clinical Global Impression Scale - Severity (CGI-S) [46] and the Children’s Global Assessment Scale (CGAS) [47] for each patient. The CGI-S is a brief clinician-rated instrument that evaluates the severity of the patient’s conditions on a scale from 1 (normal, not at all ill) to 7 (among the most extremely ill patients). The clinician also completed the Children’s Global Assessment Scale (CGAS). This scale refers to patients under 18 years old and measures the overall severity of disturbance, as a complement to specific syndrome scales. Global functioning is assessed on a scale from 0 to 100.

### Plan of statistical analyses

The sample size for a Spearman correlation was determined using power analysis. The power analysis was conducted in G*Power [48, 49] using an alpha of 0.05, a power of 0.9, and a large effect size (w = 0.8) for a two-tailed test. Because Spearman’s rank correlation coefficient is computationally identical to Pearson’s product-moment coefficient, power analysis was conducted using software for estimating power of a Pearson’s correlation. Based on the assumptions, the required sample size was determined to be 65. We conducted analyses using JASP (JASP Team, 2023. Version 0.18.1). We performed a reliability test involving Cronbach’s alpha to evaluate internal consistency. The coefficients of Cronbach’s alpha were 0.82, 0.85, and 0.80 respectively for the mothers’ TAS, fathers’ TAS, and patients’ TAS. Descriptive statistical analyses were performed for demographic and clinical characteristics of the total sample of patients and, separately, for each parent. These analyses included mean values and standard deviations (SD). Statistical comparisons were conducted between patients’ TAS scores, mothers’ TAS scores, and fathers’ TAS scores, using Spearman’s correlations. Subsequently, Spearman’s correlation was conducted for each TAS factor score (DIF, DDF, EOT), comparing patients’ scores with mothers and fathers’ scores. Both parents’ scores were also compared. Finally, Spearman’s correlation was used to compare patients’ TAS scores with CGI-S and CGAS.

## Results

The study group included 67 adolescents who suffered from REDs, together with their mothers and fathers. The average age of the patients involved was 15.2 years (SD = 1.5). Sixty-four were females and three were adolescent males. Analysing the study population, only two adolescents reported a family history of an eating disorder, while six adolescents had subthreshold criteria and 59 patients did not report a family history of an eating disorder. Only 17 parental couples were divorced or separated (25.37%). Before the diagnosis, only 36 patients had begun psychotherapy. Table 1 reported the clinical and sociodemographic characteristics of the total sample.

**Table 1:** Clinical and sociodemographic data

		Mean	SD
**Age (years)**		15.2	1.5
		**N**	**%**
**Gender at birth**	Female	64	95.5
	Male	3	4.5
		**N**	**%**
**Family history of eating disorder**	No	59	88.06
	Subthreshold	6	8.95
	Yes	2	2.9
**Diagnosis**		**N**	**%**
Neurodevelopment disorders		3	4.48
Schizophrenia/Clinical high-risk		2	2.9
Bipolar disorders		2	2.9
Depression		35	52.24
Anxiety disorders		9	13.43
Obsessive-compulsive disorders		5	7.46
Post-traumatic stress disorders		1	1.49
Dissociative disorders		0	0
Somatic symptom disorders		1	1.49
Eating disorders		67	100
Sleep disorders		0	0
Gender dysphoria		0	0
Behavioural disorders		0	0
Substance use disorders		0	0
Structuring personality disorders^a^		6	8.95

*Note*: ^a^to assess the presence of a structuring personality disorder, we administered the Structured Clinical Interview for DSM-5 Personality Disorders (SCID-5 PD) [50] to patients aged 14 and over. If the patient obtained supra-threshold scores, we gave him/her a diagnosis of structuring personality disorder since according to DSM-5 it is not correct to diagnose a personality disorder under the age of 18

Descriptive statistics have been conducted for both mothers and fathers. Family history of mental disorders has been reported for forty-two families (63%), while twenty-five families (37%) have not registered any psychiatric disorder in their family. Seventeen couples of parents (25%) were divorced, while fifty couples of parents (75%) were still together.

Descriptive statistic has been assessed for the TAS scores. In particular, the mothers’ TAS mean score was 39.28 (SD = 12.58), and the fathers’ TAS mean score was 41.09 (SD = 12.99). Patients have shown higher levels of alexithymia, with a TAS mean score of 62.25 (SD = 13.41).

**Fig. 2:**
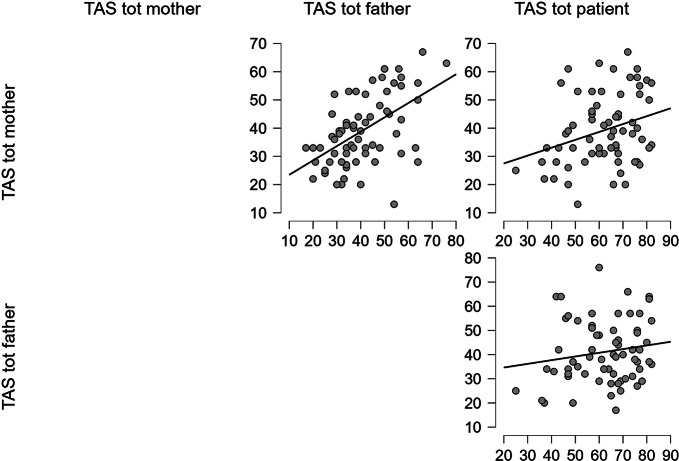
TAS total scores correlations scatter plots [Author’s own processing – JASP Version 0.18.1]

We also performed correlations between TAS total scores, as shown in Table 2; Fig. 2.

**Table 2:** Spearman’s correlation between TAS total scores in patients and their parents

Variable		TAS (mothers)	TAS (fathers)
TAS (mothers)	Spearman’s rho	-	
	*p*-value	-	
TAS (fathers)	Spearman’s rho	0.510	-
	*p*-value	< 0.001**	-
TAS (patients)	Spearman’s rho	0.269	0.137
	*p*-value	0.027*	0.270

Significance: *p < .05; **p < .001

**Fig. 3:**
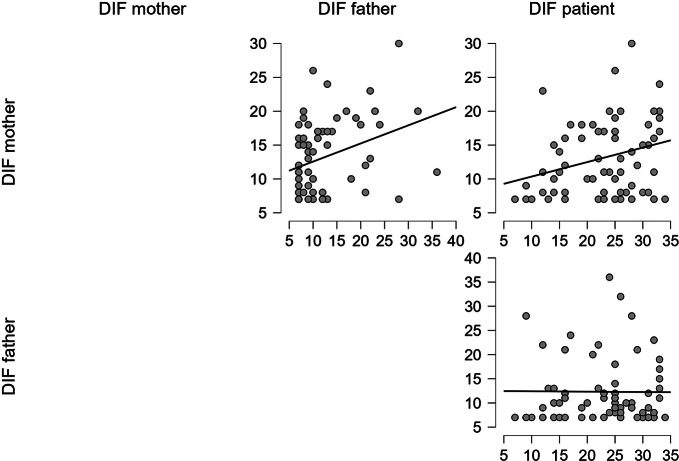
TAS DIF scores correlations scatter plots [Author’s own processing – JASP Version 0.18.1]

Considering the three alexithymia main features, there was a statistically significant correlation between patients’ DIF scores and their mothers’ scores (Spearman’s Rho = 0.276; *p* = .024); fathers’ DIF scores and mothers’ scores influence each other too (Spearman’s Rho = 0.338; *p* = .005), but this does not happen between fathers and patients’ scores (Spearman’s Rho = 0.005; *p* = .966) (Fig. 3).

**Fig. 4:**
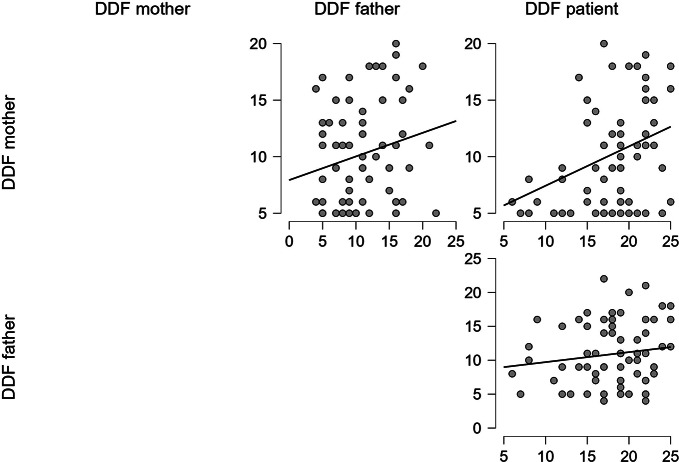
TAS DFF scores correlations scatter plots [Author’s own processing – JASP Version 0.18.1]

The statistical analyses showed a significant correlation between mothers’ and patients’ DDF scores (Spearman’s Rho = 0.325; *p =* .007) but not between mothers and fathers’ DDF scores (Spearman’s Rho = 0.219; *p* = .075), nor between fathers and patients (Spearman’s Rho = 0119; *p* = .338) (Fig. 4).

**Fig. 5:**
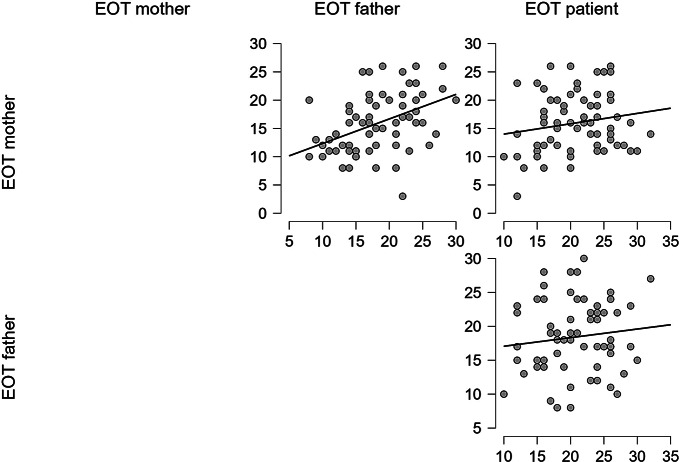
TAS EOT scores correlations scatter plots [Author’s own processing – JASP Version 0.18.1]

Finally, the mothers and patients’ EOT subscale did not show a statistically significant correlation (Spearman’s Rho = 0.164; *p* = .185) and so did the correlation between fathers and patients’ EOT scores (Spearman’s Rho = 0.098; *p* = .431), while Spearman’s correlation between mothers and fathers’ EOT scores was statistically significant (Spearman’s Rho = 0.482; *p* < .001) (Fig. 5).

The severity of the patients’ disease was assessed using CGI-S and patients’ functioning was evaluated through CGAS. The mean of the CGI-S score was 5.02 (SD = 1.06), while the mean of the CGAS score was 43.21 (SD = 13.09). No statistically significant results have been found between patients’ TAS-20 total score and CGI-S (Spearman’s Rho=-0.008; *p* = .954) nor between TAS-20 total score and CGAS (Spearman’s Rho=-0.101; *p* = .456).

## Discussion

The study focused on the relationship between patients and parents’ levels of alexithymia in families of adolescents diagnosed with REDs. The main goal of the study was to assess the alexithymic characteristics of REDs adolescents and their mothers and fathers. In particular, we aimed to evaluate if the emotional distress of RED patients might be related to their parents’ emotional functioning. The results showed the presence of a statistically significant relationship between mothers and children’s total TAS-20 scores and DIF and DDF subscales.

In the past, only a few authors deeply investigated the correlation between parents and son-daughter’s levels of alexithymia. Fonagy et al., for example, hypothesized that caregivers with emotional distress may show an uncontrolled hypersensitive response to the negative emotions of their children [51]. This difficulty may lead to an impairment in developing introspective and mentalizing abilities in children but may suggest focusing on recognizing that mothers could present emotional distress too. These difficulties may interfere both in the mother-son/daughter relationship and in the mother’s quality of life. In particular, one previous study explored the connection between family interactive patterns, showing how identifying specific triadic difficulties in family relationships may suggest the presence of serious psychopathology and underlined the requirement for an intensive and specific treatment [52]. Our findings aimed to confirm the relevance of paying attention to understanding caregivers’ difficulties in coping with their deeper emotions to provide psychological support [51].

Considering the three main alexithymia features, externally oriented thinking can be defined as people’s difficulties in exploring their inner state and focusing their energy on external events. According to previous studies, the EOT role in REDs patients is still unclear [53–55]. Analysing the different scores, it was possible to notice how only mothers and fathers’ EOT scores influence each other. Literature has demonstrated that high levels of EOT have been associated with automatic emotional processing [56]. Other authors hypothesized that patients affected by REDs show a distortion of body image, and a severe disturbance in perception, defined as “disturbance in the accuracy of perception or cognitive interpretation of stimuli arising in the body, with failure to recognize signs of nutritional need” [57] and this might be influenced by alexithymia. Mothers’ tendency to externally oriented thinking may influence fathers’ difficulties in expressing their deeper suffering. However, despite the strong relationship regarding this specific alexithymia feature that emerges in our study, no influence was found between caregivers and children’s EOT scores. Supporting this result, previous studies have underlined the low reliability of the EOT subscale. Despite the international use of TAS-20 with adolescents in previous studies, it was found that the reliability of the EOT subscale can decrease with age [14, 58]. Another factor that has to be taken into consideration is related to the nature of the externally oriented construct. This specific feature concerns the tendency not to analyse one’s own emotions: for this reason, it is possible to hypothesize that EOT scores might be difficult to capture in a precise way when this difficulty in exploring one’s inner state is not completely conscious. Analysing our results, fathers and mothers’ EOT scores seem to influence each other’s.

In the past, researchers focused on the impact of alexithymia on interpersonal relationships. Many authors discovered positive correlations between alexithymia and attachment difficulties, in particular, the tendency to place relationships as secondary and the need for more approval from others [59]. Alexithymic individuals seem to show more difficulties in creating and maintaining meaningful attachments to others. One previous study underlined a significant difference between different genders people showing how alexithymia impacts women more than men and assuming that males are socially conditioned to dwell on their inner state [60]. Despite the literature and the present results, future studies might better investigate the influence of alexithymia on couple relationships.

The present study has also revealed the influence between mothers and fathers’ difficulties in describing emotions. In general, it is possible to hypothesize that alexithymia and EOT may be traits that involve not only the patients but their parents too. Several authors have established the presence of alexithymia as a trait of personality in parents of adolescents with REDs [41]. This reduced ability to identify feelings and distinguish emotion could be considered as a reactive state to stress faced by these families, as already supposed by Freyberger [61]. Parents who present higher levels of alexithymia find it difficult to identify and describe their own emotions; as a result, they might struggle to empathize with their children’s emotions. On the other hand, some authors have already investigated the possible genetic influence on alexithymia traits [27, 28]. Considering the literature, we could assume the previous presence of alexithymia in these parents. Nevertheless, it is not possible to establish if this could influence the presence of alexithymia in their children. Further study may focus on exploring genetic correlation and the possible influence of epigenetic factors and environmental conditions on the development of difficulties in exploring one’s inner state.

Evaluating the TAS-20 score and its three main subscales, we did not find evidence of a relationship between fathers and patients’ alexithymia scores. In the past many authors focused more on mothers and their role for sons/daughters during adolescence. It is possible to hypothesize that mothers could be considered identification models for adolescents and, in particular, the primary caregiver during the ED. Despite the absence of a statistically significant impact, fathers’ emotional impairment should be taken into consideration too.

The second aim of the study was to investigate the influence of impaired emotion processing on ED severity. In particular, we hypothesize that alexithymia levels could be used as an outcome predictor. In contrast with previous studies conducted in an adult population recognizing a possible stability of alexithymia and a role as a predictor of outcome in the ED population [8, 9], no statistically significant relationship was found considering the impact of alexithymia on the severity of the disorder and patient functioning. Two previous studies did not find a precise influence of alexithymia on the outcome of these patients. Nonetheless, our results contrast with those of Speranza and colleagues, who have underlined how alexithymia may have an impact on RED outcomes [62]. It is possible to hypothesize that CGI-S and CGAS show a global overview of the ED, not evaluating more specific RED characteristics (e.g., impaired emotion processing). Future studies may consider applying specific scales such as the Eating Disorder Inventory psychological symptom scales, which focus more on emotion dysregulation and interoceptive deficits.

We also aimed to underline the complexity of the interaction between young RED patients and their families. It is possible to think that this dysfunctional interactive pattern in these families may exacerbate the alexithymic trait of patients, as previously hypothesized by Lumley and colleagues [29]. Our results confirm previous studies where alexithymia has been discovered to be the symptom of adolescent RED patients and their families who tend to avoid emotional conflicts [63]. Moreover, clinicians must consider that alexithymia can be linked to the problem of family enmeshment raised by Minuchin and colleagues [64, 65]. Enmeshment is characterized by a high degree of responsiveness and involvement, interdependence, and intrusion. Often family members struggle to differentiate perceptions of each other. So, the treatment for families of psychosomatically ill children must take into account alexithymia assessment and family functioning [42]. These findings may pave the way for future investigation of genetic and epigenetic factors in alexithymia, especially in trying to identify predictive factors for the outcome of the eating disorder. Subsequently, this may lead to the development of adequate preventive strategies to support adolescents and their families in such a delicate phase of their lives, focusing on finding individualized care pathways for young RED patients.

The findings of the current study should be considered in the context of some limitations. First, alexithymia was assessed with a self-report questionnaire. However, the TAS-20 has been chosen as it is the most widely used measure of alexithymia; it can also allow us to take into consideration another point of view of the ED, different from the clinical vision of the disorder. Finally, another limitation could be the small sample size. Future research, including a larger study population and more scales for evaluating ED, may help clarify the questions that have emerged in this study.

### Electronic supplementary material

Below is the link to the electronic supplementary material.

**Supplementary Material 1**: The RECORD statement

## Data Availability

The dataset generated and analysed during the current study is available upon request in the Zenodo repository (10.5281/zenodo.8379296).

## List of abbreviations

A-AN: atypical anorexia nervosa
AN: anorexia nervosa
ARFID: avoidant/restrictive food intake disorder
CGAS: Children’s Global Assessment Scale
CGI-S: Clinical Global Impression Scale – Severity
DDF: difficulty describing feelings
DIF: difficulty identifying feelings
ED: eating disorder
EOT: externally oriented thinking
K-SADS-PL DSM-5: Kiddie Schedule for Affective Disorders and Schizophrenia
REDs: Restrictive Eating Disorders
SCID-5-PD: Structured Clinical Interview for DSM-5 Personality Disorders
TAS-20: Toronto Alexithymia Scale
WAIS-IV: Wechsler Adult Intelligence Scale
WISC-IV: Wechsler Intelligence Scales for Children

## References

[1] Taylor GJ, Bagby RM, Parker JDA, Grotstein J. Disorders of Affect regulation [Internet]. Disorders of Affect Regulation. Cambridge University Press; 1997. [cited 2021 May 4]. Available from: /record/1997-08995-000.

[2] Martin JB, Pihl RO (1985). The stress-alexithymia hypothesis: theorectical and empirical considerations. Psychother Psychosom.

[3] Sifneos PE (1975). Problems of psychotherapy of patients with alexithymic characteristics and physical Disease. Psychother Psychosom.

[4] Hendryx MS, Haviland MG, Shaw DG (1991). Dimensions of alexithymia and their relationships to anxiety and depression. J Pers Assess.

[5] Li S, Zhang B, Guo Y, Zhang J (2015). The association between alexithymia as assessed by the 20-item Toronto Alexithymia Scale and depression: a meta-analysis. Psychiatry Res.

[6] Honkalampi K, Jokela M, Lehto SM, Kivimäki M, Virtanen M. Association between alexithymia and substance use: A systematic review and meta-analysis. Scand J Psychol [Internet]. 2022 [cited 2023 Nov 28];63(5):427–38. Available from: https://onlinelibrary.wiley.com/doi/abs/10.1111/sjop.12821. 10.1111/sjop.12821 PMC9790486 35436351

[7] Nowakowski ME, McFarlane T, Cassin S. Alexithymia and eating disorders: A critical review of the literature. J Eat Disord [Internet]. 2013 Jun 18 [cited 2021 May 4];1(1). Available from: https://pubmed.ncbi.nlm.nih.gov/24999402/. 10.1186/2050-2974-1-21 PMC4081716 24999402

[8] Gramaglia C, Gambaro E, Zeppegno P. Alexithymia and Treatment Outcome in Anorexia Nervosa: A Scoping Review of the Literature. Front Psychiatry [Internet]. 2020 Feb 14 [cited 2021 Jan 3];10:991. Available from: https://www.frontiersin.org/article/10.3389/fpsyt.2019.00991/full. 10.3389/fpsyt.2019.00991 PMC7033613 32116818

[9] Meneguzzo P, Garolla A, Bonello E, Todisco P. Alexithymia, dissociation and emotional regulation in eating disorders: Evidence of improvement through specialized inpatient treatment. Clin Psychol Psychother [Internet]. 2022 [cited 2023 Nov 28];29(2):718–24. Available from: https://onlinelibrary.wiley.com/doi/abs/10.1002/cpp.2665. 10.1002/cpp.2665 PMC9291290 34432335

[10] Mattila AK, Salminen JK, Nummi T, Joukamaa M (2006). Age is strongly associated with alexithymia in the general population. J Psychosom Res.

[11] Franz M, Popp K, Schaefer R, Sitte W, Schneider C, Hardt J (2008). Alexithymia in the German general population. Soc Psychiatry Psychiatr Epidemiol.

[12] Bagby RM, Parker JDA, Taylor GJ (1994). The twenty-item Toronto Alexithymia scale-I. item selection and cross-validation of the factor structure. J Psychosom Res.

[13] Taylor GJ, Bagby RM, Parker JDA. The 20-Item Toronto Alexithymia Scale: IV. Reliability and factorial validity in different languages and cultures. J Psychosom Res [Internet]. 2003 [cited 2021 Jun 3];55(3):277–83. Available from: https://pubmed.ncbi.nlm.nih.gov/12932803/. 10.1016/s0022-3999(02)00601-3 12932803

[14] Torres S, Guerra MP, Miller K, Costa P, Cruz I, Vieira FM (2019). Factorial Validity of the Toronto Alexithymia Scale (TAS-20) in clinical samples: a critical examination of the literature and a psychometric study in Anorexia Nervosa. J Clin Psychol Med Settings.

[15] Taylor GJ, Bagby RM, Parker JDA (2016). What’s in the name alexithymia? A commentary on ‘Affective agnosia: expansion of the alexithymia construct and a new opportunity to integrate and extend Freud’s legacy’. Neurosci Biobehav Rev.

[16] Mensi MM, Orlandi M, Rogantini C, Provenzi L, Chiappedi M, Criscuolo M et al. Assessing Family Functioning Before and After an Integrated Multidisciplinary Family Treatment for Adolescents With Restrictive Eating Disorders. Front Psychiatry [Internet]. 2021 Jun 4 [cited 2021 Jun 30];12. Available from: https://pubmed.ncbi.nlm.nih.gov/34149477/. 10.3389/fpsyt.2021.653047 PMC8211764 34149477

[17] American Psychiatric Association (2013). Diagnostic and statistical manual of mental disorders.

[18] Keski-Rahkonen A, Mustelin L. Epidemiology of eating disorders in Europe: Prevalence, incidence, comorbidity, course, consequences, and risk factors. Curr Opin Psychiatry [Internet]. 2016 Oct 1 [cited 2020 Jul 28];29(6):340–5. Available from: https://pubmed.ncbi.nlm.nih.gov/27662598/. 10.1097/YCO.0000000000000278 27662598

[19] Herpertz-Dahlmann B. Adolescent Eating Disorders: Update on Definitions, Symptomatology, Epidemiology, and Comorbidity. Child Adolesc Psychiatr Clin N Am [Internet]. 2015 [cited 2020 Dec 15];24(1):177–96. Available from: https://pubmed.ncbi.nlm.nih.gov/25455581/. 10.1016/j.chc.2014.08.003 25455581

[20] Guillén V, Santos B, Muñoz P, Fernández de Corres B, Fernández E, Pérez I et al. Toronto alexithymia scale for patients with eating disorder: Of performance using the non-parametric item response theory. Compr Psychiatry [Internet]. 2014 Jul 1 [cited 2023 Nov 28];55(5):1285–91. Available from: https://www.sciencedirect.com/science/article/pii/S0010440X14000753. 10.1016/j.comppsych.2014.03.020 24791683

[21] Westwood H, Kerr-Gaffney J, Stahl D, Tchanturia K. Alexithymia in eating disorders: Systematic review and meta-analyses of studies using the Toronto Alexithymia Scale. J Psychosom Res [Internet]. 2017 Aug [cited 2023 Nov 28];99:66–81. Available from: https://www.ncbi.nlm.nih.gov/pmc/articles/PMC5986724/. 10.1016/j.jpsychores.2017.06.007 PMC5986724 28712432

[22] Tauro JL, Wearne TA, Belevski B, Filipčíková M, Francis HM. Social cognition in female adults with Anorexia Nervosa: A systematic review. Neurosci Biobehav Rev [Internet]. 2022 Jan 1 [cited 2023 Nov 28];132:197–210. Available from: https://www.sciencedirect.com/science/article/pii/S0149763421005297. 10.1016/j.neubiorev.2021.11.035 34822877

[23] Mallorquí-Bagué N, Vintró-Alcaraz C, Sánchez I, Riesco N, Agüera Z, Granero R et al. Emotion Regulation as a Transdiagnostic Feature Among Eating Disorders: Cross-sectional and Longitudinal Approach. Eur Eat Disord Rev [Internet]. 2018 [cited 2023 Nov 28];26(1):53–61. Available from: https://onlinelibrary.wiley.com/doi/abs/10.1002/erv.2570. 10.1002/erv.2570 29168283

[24] Kench S, Irwin HJ (2000). Alexithymia and childhood family environment. J Clin Psychol.

[25] Evren C, Evren B, Dalbudak E, Ozcelik B, Oncu F (2009). Childhood abuse and neglect as a risk factor for alexithymia in adult male substance dependent inpatients. J Psychoact Drugs.

[26] Mason O, Tyson M, Jones C, Potts S (2005). Alexithymia: its prevalence and correlates in a British undergraduate sample. Psychol Psychother.

[27] Behavioral Genetics, 5th Edition, Plomin R, DeFries JC, Gerald E, McClearn P, McGuffin. New York, NY: Worth Publishers, ISBN10: 1-4292-0577-6, ISBN13: 978-1-4292-057, XVIII introductory pages, 505 text pages. Intelligence. 2008;36:732–3.

[28] Picardi A, Fagnani C, Gigantesco A, Toccaceli V, Lega I, Stazi MA. Genetic influences on alexithymia and their relationship with depressive symptoms. J Psychosom Res [Internet]. 2011 Oct 1 [cited 2023 Nov 28];71(4):256–63. Available from: https://www.sciencedirect.com/science/article/pii/S002239991100095X. 10.1016/j.jpsychores.2011.02.016 21911104

[29] Lumley MA, Mader C, Gramzow J, Papineau K (1996). Family factors related to alexithymia characteristics. Psychosom Med.

[30] Godart N, Berthoz S, Curt F, Perdereau F, Rein Z, Wallier J et al. A randomized controlled trial of adjunctive family therapy and treatment as usual following inpatient treatment for anorexia nervosa adolescents. PLoS ONE [Internet]. 2012 Jan 4 [cited 2020 Jun 22];7(1). Available from: https://pubmed.ncbi.nlm.nih.gov/22238574/. 10.1371/journal.pone.0028249 PMC3251571 22238574

[31] Martini MG, Barona-Martinez M, Micali N. Eating disorders mothers and their children: a systematic review of the literature. Arch Womens Ment Health [Internet]. 2020 Aug 1 [cited 2023 Nov 28];23(4):449–67. 10.1007/s00737-020-01019-x. 10.1007/s00737-020-01019-x PMC7368867 31938867

[32] Bauer KW, Bucchianeri MM, Neumark-Sztainer D. Mother-reported parental weight talk and adolescent girls’ emotional health, weight control attempts, and disordered eating behaviors. J Eat Disord [Internet]. 2013 Dec 27 [cited 2023 Nov 28];1(1):45. 10.1186/2050-2974-1-45. 10.1186/2050-2974-1-45 PMC4081797 24999423

[33] Lantzouni E, Cox MH, Salvator A, Crosby RD (2015). Mother-daughter coping and disordered eating. Eur Eat Disord Rev J Eat Disord Assoc.

[34] Loth KA, MacLehose RF, Fulkerson JA, Crow S, Neumark-Sztainer D (2014). Are food restriction and pressure-to-eat parenting practices associated with adolescent disordered eating behaviors?. Int J Eat Disord.

[35] Wallis A, Miskovic-Wheatley J, Madden S, Rhodes P, Crosby RD, Cao L et al. How does family functioning effect the outcome of family based treatment for adolescents with severe anorexia nervosa? J Eat Disord [Internet]. 2017 Dec 13 [cited 2020 Dec 17];5(1). Available from: /pmc/articles/PMC5729267/?report = abstract. 10.1186/s40337-017-0184-9 PMC5729267 29255605

[36] Rienecke RD, Accurso EC, Lock J, Le Grange D, Expressed, Emotion. Family Functioning, and Treatment Outcome for Adolescents with Anorexia Nervosa. Eur Eat Disord Rev J Eat Disord Assoc [Internet]. 2016 Jan [cited 2023 Nov 28];24(1):43–51. Available from: https://www.ncbi.nlm.nih.gov/pmc/articles/PMC4962527/. 10.1002/erv.2389 PMC4962527 26201083

[37] Rienecke RD, Lebow J, Lock J, Le Grange D. Family Profiles of Expressed Emotion in Adolescent Patients With Anorexia Nervosa and Their Parents. J Clin Child Adolesc Psychol [Internet]. 2017 May 4 [cited 2023 Nov 28];46(3):428–36. 10.1080/15374416.2015.1030755. 10.1080/15374416.2015.1030755 25945418

[38] Baradel G, Provenzi L, Chiappedi M, Orlandi M, Vecchio A, Borgatti R et al. The Family Caregiving Environment Associates with Adolescent Patients’ Severity of Eating Disorder and Interpersonal Problems: A Cross-Sectional Study. Children [Internet]. 2023 Feb [cited 2023 Feb 3];10(2):237. Available from: https://www.mdpi.com/2227-9067/10/2/237. 10.3390/children10020237 PMC9955592 36832366

[39] Suslow T, Donges US (2017). Alexithymia Components are differentially related to Explicit negative Affect but Not Associated with Explicit positive affect or implicit affectivity. Front Psychol.

[40] Guttman H, Laporte L (2002). Alexithymia, empathy, and psychological symptoms in a family context. Compr Psychiatry.

[41] Espina A. Alexithymia in parents of daughters with eating disorders: Its relationships with psychopathological and personality variables. J Psychosom Res [Internet]. 2003 Dec 1 [cited 2023 Nov 28];55(6):553–60. Available from: https://www.sciencedirect.com/science/article/pii/S0022399903000163. 10.1016/s0022-3999(03)00016-3 14642987

[42] Coci C, Provenzi L, De Giorgis V, Borgatti R, Chiappedi M, Mensi MM et al. Family Dysfunctional Interactive Patterns and Alexithymia in Adolescent Patients with Restrictive Eating Disorders. Child 2022 Vol 9 Page 1038 [Internet]. 2022 Jul 12 [cited 2022 Aug 10];9(7):1038. Available from: https://www.mdpi.com/2227-9067/9/7/1038/htm. 10.3390/children9071038 PMC9323591 35884021

[43] Kaufman J, Birmaher B, Axelson D, Pereplitchikova F, Brent D, Ryan N (2016). Schedule for affective disorders and Schizophrenia for School-aged children: Present and Lifetime Version (K-SADS-PL) DSM-5.

[44] Wechsler D. Wechsler Intelligence Scale for Children-Fourth edition (WISC-IV). Giunti Organizzazioni Speciali, editor. Firenze; 2003.

[45] Wechsler D. Wechsler Adult Intelligence Scale-Fourth edition (WAIS-IV). Giunti Organizzazioni Speciali, editor. Firenze; 2008.

[46] Guy W. ECDEU: Assessment Manual for Psychopharmacology (revised). Vol. 1, Nimh. Rockville, MD: U.S. Department of Health, Education, and Welfare; 1976. 217–221 p.

[47] Shaffer D, Gould MS, Brasic J, Ambrosini P, Fisher P, Aluwahlia S et al. A Children’s Global Assessment Scale (CGAS). Arch Gen Psychiatry [Internet]. 1983 Nov 1 [cited 2020 Apr 5];40(11):1228–31. Available from: https://pubmed.ncbi.nlm.nih.gov/6639293/. 10.1001/archpsyc.1983.01790100074010 6639293

[48] Faul F, Erdfelder E, Lang AG, Buchner A. G*Power 3: A flexible statistical power analysis program for the social, behavioral, and biomedical sciences. Behav Res Methods [Internet]. 2007 May 1 [cited 2023 Oct 15];39(2):175–91. 10.3758/BF03193146. 10.3758/bf03193146 17695343

[49] Faul F, Erdfelder E, Buchner A, Lang AG. Statistical power analyses using G*Power 3.1: Tests for correlation and regression analyses. Behav Res Methods [Internet]. 2009 Nov 1 [cited 2023 Oct 15];41(4):1149–60. 10.3758/BRM.41.4.1149. 10.3758/BRM.41.4.1149 19897823

[50] First MB, Williams JBW, Janet SB, Spitzer RL. Structured clinical interview for DSM-5. Personality disorders (SCID-5-PD). American Psychiatric Association; 2017.

[51] Fonagy P, Target M (1995). Understanding the violent patient: the use of the body and the role of the father. Int J Psychoanal.

[52] Balottin L, Mannarini S, Mensi MM, Chiappedi M, Balottin U. Are family relations connected to the quality of the outcome in adolescent anorexia nervosa? An observational study with the Lausanne Trilogue Play. Clin Psychol Psychother [Internet]. 2018;25(6):785–96. Available from: https://onlinelibrary.wiley.com/doi/abs/10.1002/cpp.2314. 10.1002/cpp.2314 30051637

[53] Gramaglia C, Ressico F, Gambaro E, Palazzolo A, Mazzarino M, Bert F et al. Alexithymia, empathy, emotion identification and social inference in anorexia nervosa: A case-control study. Eat Behav [Internet]. 2016 Aug 1 [cited 2021 Jun 3];22:46–50. Available from: https://linkinghub.elsevier.com/retrieve/pii/S1471015316300393. 10.1016/j.eatbeh.2016.03.028 27086047

[54] Torres S, Guerra MP, Lencastre L, Roma-Torres A, Brandão I, Queirós C et al. Cognitive processing of emotions in anorexia nervosa. Eur Eat Disord Rev [Internet]. 2011;19(2):100–11. 10.1002/erv.1046. 10.1002/erv.1046 20928928

[55] Montebarocci O, Codispoti M, Baldaro B, Rossi N (2004). Adult attachment style and alexithymia. Personal Individ Differ.

[56] Espina Eizaguirre A, Ortego Saenz de Cabezón A, Ochoa de Alda I, Joaristi Olariaga L, Juaniz M. Alexithymia and its relationships with anxiety and depression in eating disorders. Personal Individ Differ [Internet]. 2004;36(2):321–31. Available from: https://linkinghub.elsevier.com/retrieve/pii/S0191886903000990.

[57] Bruch H (1962). Perceptual and conceptual disturbances in Anorexia Nervosa. Psychosom Med.

[58] Loas G, Braun S, Delhaye M, Linkowski P (2017). The measurement of alexithymia in children and adolescents: psychometric properties of the Alexithymia Questionnaire for Children and the twenty-item Toronto Alexithymia Scale in different non-clinical and clinical samples of children and adolescents. PLoS ONE.

[59] Hesse C, Floyd K. Affection mediates the impact of alexithymia on relationships. Personal Individ Differ [Internet]. 2011 Apr 1 [cited 2023 Nov 28];50(4):451–6. Available from: https://www.sciencedirect.com/science/article/pii/S0191886910005428.

[60] Onnis L, Di Genaro A (1987). Alexitimia: una revisione critica (Alexithymia: a critical review). Med Psicosom.

[61] Freyberger H. Supportive Psychotherapeutic Techniques in Primary and Secondary Alexithymia / Discussion. Psychother Psychosom [Internet]. 2010 Feb 16 [cited 2023 Nov 28];28(1–4):337–45. 10.1159/000287080. 10.1159/000287080 609693

[62] Speranza M, Loas G, Wallier J, Corcos M. Predictive value of alexithymia in patients with eating disorders: A 3-year prospective study. J Psychosom Res [Internet]. 2007 Oct [cited 2021 May 4];63(4):365–71. Available from: https://pubmed.ncbi.nlm.nih.gov/17905043/. 10.1016/j.jpsychores.2007.03.008 17905043

[63] Balottin L, Nacinovich R, Bomba M, Mannarini S (2014). Alexithymia in parents and adolescent anorexic daughters: comparing the responses to TSIA and TAS-20 scales. Neuropsychiatr Dis Treat.

[64] Minuchin S, Baker L, Rosman BL, Liebman R, Milman L, Todd TC. A Conceptual Model of Psychosomatic Illness in Children: Family Organization and Family Therapy. Arch Gen Psychiatry [Internet]. 1975 [cited 2020 Dec 17];32(8):1031–8. Available from: https://pubmed.ncbi.nlm.nih.gov/808191/. 10.1001/archpsyc.1975.01760260095008 808191

[65] Minuchin S, Rosman BL, Baker L, Liebman R. Psychosomatic families [Internet]. Psychosomatic families. Harvard University Press; 2014. [cited 2020 Jul 28]. Available from: /record/1979-24094-000.

